# TAILcaller: an R package for analyzing differences in poly(A) tail length for Oxford Nanopore RNA sequencing

**DOI:** 10.1093/bioadv/vbaf235

**Published:** 2025-09-26

**Authors:** Mateusz Maździarz, Łukasz Paukszto, Jakub Sawicki

**Affiliations:** Department of Botany and Evolutionary Ecology, University of Warmia and Mazury in Olsztyn, Olsztyn 10-719, Poland; Department of Botany and Evolutionary Ecology, University of Warmia and Mazury in Olsztyn, Olsztyn 10-719, Poland; Department of Botany and Evolutionary Ecology, University of Warmia and Mazury in Olsztyn, Olsztyn 10-719, Poland

## Abstract

**Motivation:**

Changes in poly(A) tail length were identified as a key post-transcriptional mechanism that controls protein synthesis. The length of the poly(A) tail was shown to directly impact mRNA stability and translation, broadening its regulatory scope. A thorough understanding of poly(A) tail dynamics is considered essential for deciphering how genes are regulated and what role they play in cellular function. Therefore, we developed *TAILcaller* to empower researchers to perform analyses based on poly(A) tail length. *TAILcaller* is particularly useful for researchers utilizing nanopore sequencing, as it allows for direct analysis of data from BAM files generated by *dorado*, eliminating the need for complex scripts and intermediate tools. *TAILcaller* supports direct RNA sequencing data generated by *dorado* (—estimate-poly-a) and cDNA sequencing reads processed analogously.

**Results:**

The *TAILcaller* program was utilized to investigate changes in poly(A) tail lengths in response to nanoplastic exposure and in *Riccia fluitans* under varying environmental conditions. Significant differences in poly(A) tail lengths were revealed across different nanoplastic concentrations, with distinct patterns being observed between control and exposed groups. Furthermore, a global elongation in poly(A) tail lengths was noted in the aquatic environment compared to the terrestrial environment in *R. fluitans*. Numerous genes with differential poly(A) tail lengths were effectively identified by *TAILcaller*’s adaptive statistical functions, thereby demonstrating its utility in detecting subtle biological responses.

**Availability and implementation:**

*TAILcaller* is available at https://github.com/Mordziarz/TAILcaller.

## 1 Introduction

The 3′ poly(A) tail of mRNA is a key regulator of gene expression, dynamically responding to diverse cellular cues. Variations in its length serve as a crucial post-transcriptional mechanism for controlling protein synthesis (Kühn and Wahle 2004). This flexibility is vital for cellular adaptation to stress, environmental changes, and cell cycle progression (Sachs 1993). Stress responses, environmental shifts, and cell cycle transitions induce rapid poly(A) tail length changes, enabling swift gene expression modulation (Mangus *et al.* 2003). Tail length impacts mRNA stability and translation, expanding its regulatory reach (Decker and Parker 1994). Interactions with poly(A)-binding proteins (PABPs) underpin this regulatory network (Kühn and Wahle 2004). Understanding poly(A) tail dynamics is essential for deciphering gene regulation and its role in cellular functions (Sandberg *et al.* 2008).

Poly(A) tail length analysis has become a widely used method for studying post-transcriptional regulation, in plants (Kappel *et al.* 2015, Jia *et al.* 2022, Maździarz *et al.* 2024) and animals (Viscardi and Arribere 2022, Biziaev *et al.* 2024, Czarnocka-Cieciura *et al.* 2025). The poly(A) tail is not a static, straightforward unit that merely signifies the 3′ end of a transcript; instead, the poly(A) tail is regarded as a dynamic and variable part of the transcript (Jalkanen *et al.* 2014). In plants and animals, median poly(A) tail lengths have been observed to range from 50 to 100 nucleotides, with tissue-specific variations (Chang *et al.* 2014, Jia *et al.* 2022). Interestingly, a correlation between poly(A) tail length and mRNA half-life has been found, but no correlation with translational efficiency (Chang *et al.* 2014).

Continuous efforts are being made by Oxford Nanopore Technologies (ONT) to improve its technology and facilitate the analysis of sequencing data. The guppy basecaller has been replaced by *dorado*, and fast5 files have been replaced by pod5, just two of many changes implemented in recent years. Furthermore, the new direct RNA sequencing chemistry, SQK-RNA004, has rendered older models, such as those implemented in nanopolish (https://github.com/jts/nanopolish), less effective, requiring them to be retrained.

Nanopore sequencing, combined with the precise basecaller *dorado* (https://github.com/nanoporetech/dorado), can be used to investigate differences in poly(A) tail length, enabling the estimation of poly(A) tail lengths in transcripts. The development of a bioinformatics tool is necessary to facilitate the extraction of poly(A) tail length information from BAM files after basecalling with *dorado*, and to aid in their subsequent analysis. With *dorado* updates, the estimation of poly(A) tail lengths has been made possible by adding the—estimate-poly-a flag to the basecaller command. Previously, programs like nanotail (https://github.com/smaegol/nanotail), which is still in its developmental phase and the ONT pipeline (https://github.com/nanoporetech/pipeline-polya-diff/) were used for poly(A) tail analysis based on nanopolish and the guppy basecaller.

The main challenge lies in extracting poly(A) tail length information from BAM files after *dorado* basecalling, followed by performing differential analyses and visualizing the results. To address this challenge, *TAILcaller* was created, an R library for analyzing BAM files from *dorado* basecalling using the—reference and—estimate-poly-a flags, designed to simplify poly(A) tail analysis. *TAILcaller* is compatible with direct RNA sequencing data generated by *dorado* (—estimate-poly-a), as well as cDNA sequencing reads processed similarly.

## 2 Methods

### 2.1 Plant material, RNA extraction, and cDNA sequencing

Plants were grown *in vitro*, following the method described in our previous works (Sawicki *et al.* 2023, Maździarz *et al.* 2024). After this period, the plants for 2 weeks were exposed to a suspension containing nanoplastic particles at concentrations of 0.1 mg/l (LOW) and 10 mg/l (HIGH). The control group (CTR) was flooded without nanoplastic. After 2 weeks, the plants were harvested, and their thalli were ground in a mortar with liquid nitrogen. Total RNA was then extracted using the Qiagen RNeasy Plant Mini Kit, following the manufacturer’s instructions. RNA concentration was assessed using Qubit RNA HS Assay Kit and Qubit fluorometer. Sequencing libraries for nanopore sequencing were prepared with PCR-cDNA Barcoding Kit SQK-PCB 111.24, using 300 ng of total RNA for each sample. The protocol included the following steps: cDNA-RT adapter ligation, adapter digest, reversed transcription, strand switching, barcode incorporation, PCR with rapid attachment primers, attachment of rapid sequencing adapters. Library constructs were loaded on previously primed Promethion Flow Cell R10.4.1 and sequenced with PromethION 2 Solo sequencing unit.

### 2.2 Plant material, RNA extraction, and direct RNA sequencing

As in the previous case, the *Riccia fluitans (R*.* fluitans)* RF1 line, which was used as the plant material, was sourced from an established axenic *in vitro* culture. Total RNA was subsequently extracted using the RNA Plant mini spin kit (Qiagen), strictly adhering to the manufacturer’s protocol. Both RNA quality and quantity were meticulously assessed: the former was evaluated using a Tapestation (Agilent) equipped with the High sensitivity RNA screening tape kit, and the latter was determined with a Qubit 4 fluorometer utilizing the HS RNA assay kit. For long-read native RNA sequencing, libraries were meticulously prepared from 50 ng of poly(A)-tailed mRNA per sample. This process was carried out using the direct RNA sequencing kit SQK-RNA002 ONT. To remove rRNA from total RNA, NEBNext Poly(A) mRNA Magnetic Isolation Module (New England Biolabs) was used. Following this, SuperScript III reverse transcriptase (Thermo Fisher Scientific) was employed for the synthesis of the second cDNA strand, facilitating the formation of RNA-cDNA hybrids. Subsequently, sequencing adapters were ligated using T4 DNA ligase (New England Biolabs). Finally, the prepared libraries were quantified with the Qubit dsDNA HS assay kit (ThermoFisher). Sequencing was performed on a MinION MK1C device (ONT), utilizing FLO-MIN 106 Flow Cells R.9.4.1 (ONT), which had been prepared beforehand with the flow cell priming kit EXP-FLP002 (ONT).

### 2.3 Transcriptome preparation in *R. fluitans*

Nanopore cDNA sequencing pod5 files were basecalled using *dorado* v.0.8.1. The resulting BAM files were then converted to FASTQ files with *bedtools* v.2.30.0 (Quinlan and Hall 2010). These FASTQ files were subsequently mapped to the *R*. *fluitans* genome (Levins *et al.* 2025) using *minimap2* v.2.27, employing the -ax splice flag (Li 2018). Finally, these BAM files were used to assemble the transcriptome with *stringtie* v.3.0.0 (Shumate *et al.* 2022) and gffread v.0.12.7 (Pertea and Pertea 2020).

### 2.4 Poly(A) tail analysis

Following transcriptome preparation, basecalling was repeated with *dorado* on both cDNA and direct RNA sequencing POD5 files. This secondary basecalling was performed using the sup option along with the—reference and—estimate-poly-a flags. All BAM files were deposited at the following link: https://doi.org/10.6084/m9.figshare.28632515.v3. *TAILcaller* v.0.2.0 was then used to analyze differences in poly(A) tails across two distinct projects: one investigating the impact of nanoplastics on *R. fluitans*, and another comparing poly(A) tail lengths between aquatic(W) and terrestrial(L) *R. fluitans* specimens. For analysis at the gene level, genes with fewer than 10 poly(A) tails were excluded. Genes were considered as differentially polyadenylated genes (DPGs) if their poly(A) tail length fluctuations were found to be significantly different with an adjusted *P* value (*P*.adj) of less than .05.

### 2.5 Implementation

**Figure 1.**
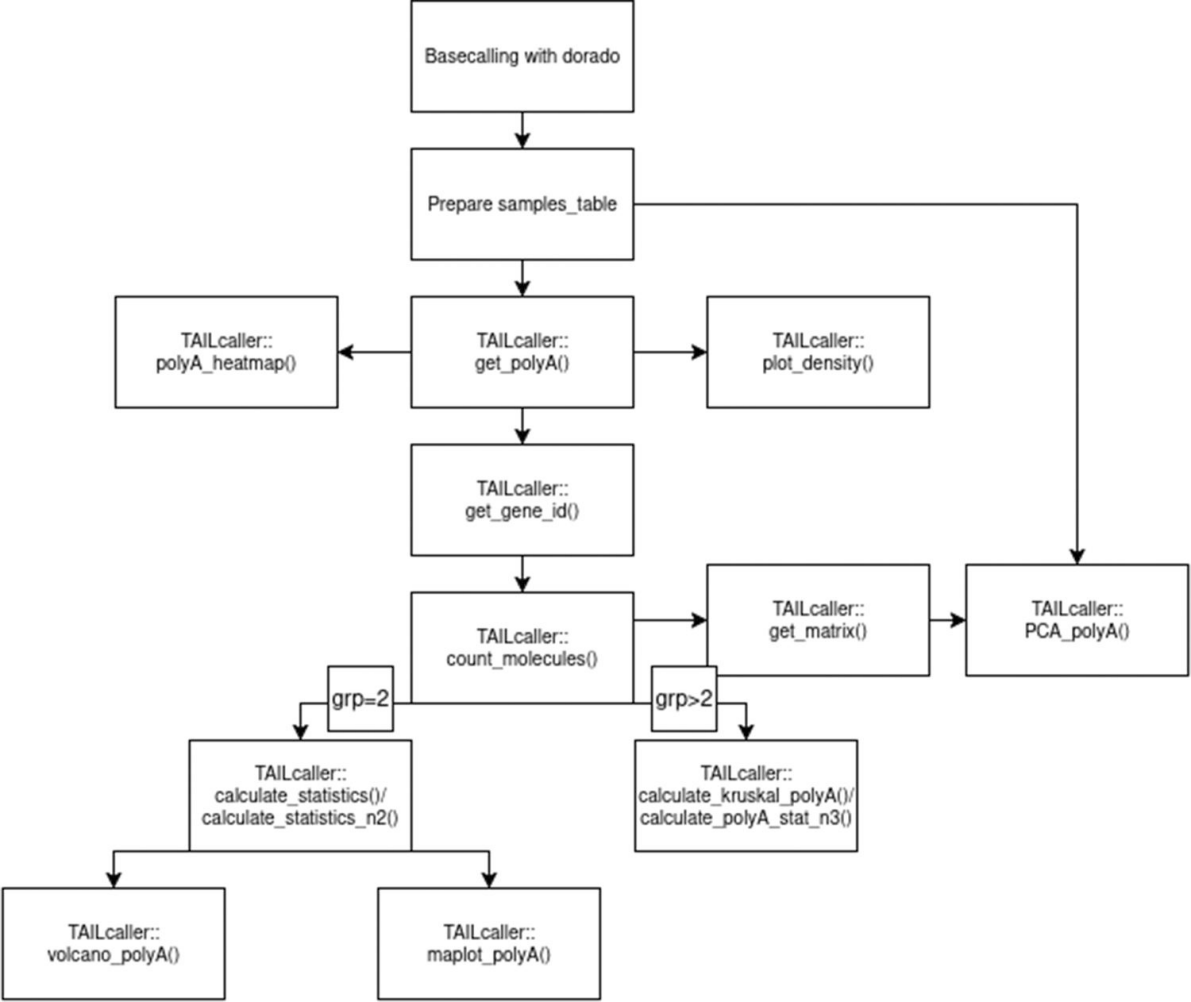
Flowchart showing the *TAILcaller* operations. The process begins with basecalling using *dorado* software, followed by data preparation within a “samples_table.” Subsequently, poly(A) tail extraction (*TAILcaller*::get_polyA()) and gene identification (*TAILcaller*::get_gene_id()) are performed. Concurrently, heatmap (*TAILcaller*::polyA_heatmap()) and density plot (*TAILcaller*::plot_density()) of poly(A) tails are generated. The number of poly(A) molecules (*TAILcaller*::count_molecules()) is then analyzed. For two groups (grp = 2), statistical calculations (*TAILcaller*::calculate_statistics()/calculate_statistics_n2()) are conducted, leading to the generation of volcano plots (*TAILcaller*::volcano_polyA()) and MA plots (*TAILcaller*::maplot_polyA()). When more than two groups are present (grp > 2), Kruskal-Wallis, ANOVA and Welch ANOVA tests (*TAILcaller*::calculate_Kruskal_polyA()/*TAILcaller*::calculate_polyA_stat_n3()) are applied. Additionally, principal component analysis (*TAILcaller*::PCA_polyA()) is performed on the poly(A) data derived from the data matrix (*TAILcaller*::get_matrix()).

In Fig. 1, the summary of the *TAILcaller* workflow is illustrated. Analysis is enabled by *TAILcaller* through information extraction from a BAM file, which is generated after basecalling with *dorado*, utilizing the --reference and --estimate-poly-a flags. The *TAILcaller* package had to be installed and loaded into the R environment using the devtools package. Detailed installation instructions can be found on the *TAILcaller* GitHub page (https://github.com/Mordziarz/TAILcaller). A test dataset and script were provided to allow for quick testing of the package. The package was built using the following R libraries: *Rsamtools* v.2.20.0 (Li *et al.* 2009), *dplyr* v.1.1.4, *stats* v.4.4.1, tidyr v.1.3.1, *rlang* v.1.1.5, *ggplot2* v.3.5.1 (Villanueva and Chen 2019), *circlize* v.0.4.16 (Gu *et al.* 2014), *ComplexHeatmap* v.2.20.0 (Gu *et al.* 2016), *ggtree* v.3.12.0 (Yu et al. 2017), *car* v.3.1, *dunn.test* v.1.3.6, *nortest* v.1.0, and *rstatix* v.0.7.2.

**“get_polyA()”**—BAM files are processed after basecalling with *dorado* using the—estimate-poly-a and –reference flags. The “samples_table” argument is accepted, which constitutes a table containing BAM files and their descriptions. A single table with columns “read_id,” “transcript_id,” “polyA_length,” “sample_name,” and “group” (n) is outputted by the function. BAM file scanning is enabled through the *Rsamtools*::ScanBamParam() and *Rsamtools*::scanBam() functions.

**“density_plot()”—**Density visualization is performed using the *ggplot2*::ggplot() function, and statistical analysis between groups is conducted. The arguments “polyA_table” (table resulting from the “get_polyA()” function), “stats” (which can accept “mean” or “median,” representing horizontal dashed lines on the density plot), and “grouping_factor” (column name from the polyA_table such as “sample_name” or “group”) are accepted. Statistical differences are calculated between the grouping factors. For two groups (grp = 2): When normal distribution is exhibited in both groups (*stats*::shapiro.wilk() or *nortest*::lillie.test()) and homogeneous variances are present (*car*::leveneTest()), a two-sample Student’s *t*-test is performed (*stats*::t.test()). When normal distribution is exhibited but unequal variances are present, Welch’s *t*-test is conducted (*stats*::t.test()). Otherwise (non-normality, unequal variances, or fewer than three observations in any group), a Wilcoxon rank-sum test (*stats*::wilcox.test()) is performed. For more than two groups (grp > 2): When normal distribution is shown in all groups and homogeneous variances are present, one-way analysis of variance (*stats*::aov()) is applied, followed by Tukey’s HSD *post-hoc* test (*stats*::TukeyHSD()) for pairwise comparisons. When normality is maintained but variances are unequal, Welch’s ANOVA (*stats*::oneway.test()) and Games-Howell test (*rstatix*::games_howell_test()) are performed. Otherwise (non-normality or fewer than three observations in any group), a Kruskal-Wallis test (*stats*::kruskal.test()) is executed, coupled with Dunn’s multiple comparisons (*dunn.test*::dunn.test()) using Bonferroni correction.

**“get_gene_id()”**—A table created by the “get_polyA()” function is merged with gene identifiers extracted from a GTF (Gene Transfer Format) file through the “polyA_table” and “gtf_file” arguments. The merge is performed by matching transcript IDs (defined in “transcript_column_gtf”) between the two datasets, enabling gene-level analysis of poly(A) tail length data. Additionally, the column from the GTF containing gene identifiers must be defined in the “gene_column_gtf” argument.

**“polyA_duplicates()”—**Function is used to assess the uniqueness and multiplicity of “read_id” assigned to transcripts. Within this function, “read_id” are checked to determine how many are uniquely assigned, duplicated, or multiply replicated. A straightforward bar plot is then generated using *ggplot2*::ggplot() to visually represent the distribution of these unique, duplicated, or multiply replicated “read_id”.

**“count_molecules()”—**The number of molecules is counted at the transcript level (“which_level” = column with transcripts) or the gene level (“which_level” = column with genes) in groups (“grouping_factor”).

**“calculate_statistics()”—**This function calculates *P* value significance for genes/transcripts using the Wilcoxon test (*stats*::wilcox.test()), considering the which_level and grouping_factor specifications. A polyA_table must be supplied, which can be the output of “get_polyA()” (for transcript-level analysis) or “get_gene_id()” (for gene-level analysis). The control_group and treated_group must be designated. *P* value adjustment is supported, offering methods like “holm,” “Hochberg,” “hommel,” “Bonferroni,” “BH,” “BY,” and “fdr” through *stats*::p.adjust(). Furthermore, Cohen’s *d* test and log2foldchange values are also derived.

**“calculate_statistics_n2()”—**This function takes the same inputs as calculate_statistics(), but it offers a more flexible approach to *P* value calculation than just the Wilcoxon test. Specifically, if both groups show normal distribution (verified using *stats*::shapiro.test() or *nortest*::lillie.test()) and exhibit similar variances (assessed by *car*::leveneTest()), a two-sample Student’s *t*-test (*stats*::t.test()) is performed. Should normal distribution be present but variance heterogeneity exists, Welch’s *t*-test (*stats*::t.test()) is applied instead. In all other cases—meaning if normality is not met, variances are unequal, or any group has fewer than 3 observations—the Wilcoxon rank-sum test (*stats*::wilcox.test()) is conducted.

**“calculate_kruskal_polyA()”—** *P* value significance is calculated using the Kruskal-Wallis test (*stats*::kruskal.test()) for genes/transcripts depending on the defined “which_level” between the grouping column defined in “grouping_factor.” The “polyA_table” must be provided, which can be a table directly obtained from the “get_polyA()” function (for transcript analysis) or after the “get_gene_id()” function (for gene analysis). *P* value correction can be performed using the same methods as in “calculate_statistics()”.

**“calculate_polyA_stat_n3()”—**The same arguments as “calculate_kruskal_polyA()” are accepted. However, *P* value calculation is not restricted to the Kruskal-Wallis test alone. When normality is confirmed in all groups and variance homogeneity is established, one-way analysis of variance (*stats*::aov()) is implemented. When normality is preserved but variance heterogeneity is detected, Welch’s ANOVA (*stats*::oneway.test()) is executed. In all other scenarios (non-normality or fewer than three observations in any group), a Kruskal-Wallis test (*stats*::kruskal.test()) is performed. *P* value correction is performed in the same manner as in “calculate_statistics()”.

**“volcano_polyA()”—**A publication-ready volcano plot is generated via the *ggplot2*::ggplot() function. This visualization’s design effectively conveys changes: −log10(padj) values are mapped to the Y-axis, while log2FoldChange for a given gene or transcript is presented on the X-axis. The function requires the calculate_statistics_out argument, which expects the table produced by the “calculate_statistics()” or “calculate_statistics_n2()” functions. Furthermore, “collapsed_color” is utilized to assign the color for data points representing significant genes/transcripts that demonstrate a shortening of poly(A) tail length between the two compared groups. Conversely, “expansion_color” serves to define the color for genes/transcripts where an extension in poly(A) tail length is observed.

**“maplot_polyA()”—**A publication-ready MA plot can be generated using the *ggplot2*::ggplot() function, which illustrates changes in log2FoldChange on the Y-axis and mean poly(A) tail values for genes/transcripts on the X-axis. The arguments “calculate_statistics_out” (table obtained after “calculate_statistics()” or “calculate_statistics_n2()” functions), “collapsed_color” (color to be assigned to points on the plot for significant genes/transcripts whose tail length shortens between two groups), and “expansion_color” (for genes/transcripts where tail length is longer) are accepted.

**“get_matrix()”—**A matrix can be created based on the table from the “count_molecules()” function. The statistics can be “count” (expression), “avg_polyA_length” (mean), or “median_polyA_length” (median). If values for transcripts are to be counted (“which_level”=“transcript_id”), this should be specified. If counting for genes is desired, “which_level”=“gene_id” should be used.

**“PCA_polyA()”—**Principal component analysis (PCA) can be performed. The arguments “get_matrix_out” (resulting table from the “get_matrix” function), “samples_table” (initial table with BAM files and their descriptions), and “grouping_factor” (column from the samples_table that will constitute the grouping factor) are accepted. The plot representing PCA is created using the *ggplot2*::ggplot() function, while the PCA analysis itself is enabled through the *stats*::prcomp().

**“polyA_heatmap()”—**Poly(A) tail lengths can be visualized as a heatmap and hierarchical clustering is performed. The arguments “polyA_table” (resulting table from the “get_polyA()” function), “grouping_factor” (grouping factor—column from polyA_table), “frame” (window for counting tail lengths, e.g. 10 creates windows 1–10, 11–20, 21–30, etc.), “select” (which can accept “base” for actual poly(A) tail lengths or “normalized” for percentage content of poly(A) tail lengths), and “heatmap_color” (accepting values “red_green,” “green_red,” “blue_green,” “green_blue,” “blue_red,” “red_blue” representing different heatmap color palettes encoded through the *circlize*::colorRamp2() function) are accepted. The heatmap and hierarchical clustering are obtained through the *ComplexHeatmap*::Heatmap() and *ggtree*::ggtree() functions.

### 2.6 Navigating challenges and overcoming errors

A primary limitation, when *TAILcaller* was used, involved the inability for gene/transcript-specific analysis to be performed after a genome had been defined in *dorado*’s—reference argument. Once the genome was defined, users were able to conduct a general analysis comparing global poly(A) tail length distributions. However, direct gene/transcript-level analysis from the *dorado* BAM file was not possible. Instead, reads had to be manually assigned to transcripts, and this information then had to be manually added to the output table that was generated by the *TAILcaller*::get_polyA() function. Therefore, the future addition of a function within *TAILcaller* that assigns transcripts to reads was considered a significant challenge.

Although a count matrix was produced for use by expression analysis programs following the execution of the *TAILcaller::*get_matrix() function, the automation of the differential expression analysis process was not yet implemented. It was proposed that a function be developed to accept the count matrix produced by *TAILcaller*::get_matrix() and perform the differential expression analysis (Table 1, available as supplementary data at *Bioinformatics Advances* online).

It was possible for a single “read_id” to be assigned to two transcripts. Specifically for this problem, the *TAILcaller*::polyA_duplicates() function was added, which allowed users to remove these duplications. In most cases, users had to decide independently which transcript corresponded to which “read_id.” However, users were often unable to determine this even by manual checking. In the case of analyses at the transcript level, it was recommended to leave these duplicates if it was not possible to resolve which transcript a given “read_id” belonged to. Conversely, when analysis was performed at the gene level, it was recommended that duplicates be removed (specifically, duplicated transcripts with the same poly(A) tail length within one gene), as results could be artificially skewed by their presence.

An interesting case for future research was considered to be poly(A) tail length, which could serve as a factor for determining similarities and differences between organisms. The *TAILcaller*::polyA_heatmap() function was programmed to enable the creation of a heatmap and a dendrogram (phylogenetic tree). Information regarding similarities and differences between plants was also provided by poly(A) tail lengths (Jia *et al.* 2022). Dendrograms, which were based on hierarchical clustering, were constructed using Relative Synonymous Codon Usage values (Behura and Severson, 2012, Ciborowski *et al.* 2024, Maździarz *et al.* 2025) and these provided additional information regarding relationships between organisms. It was thought that this approach, which utilized hierarchical clustering based on poly(A) tail lengths, could be a subject of future research for identifying another factor of similarity between organisms.

## 3 Results

### 3.1 Demonstrating *TAILcaller*: insights from nanoplastic exposure

**Figure 2.**
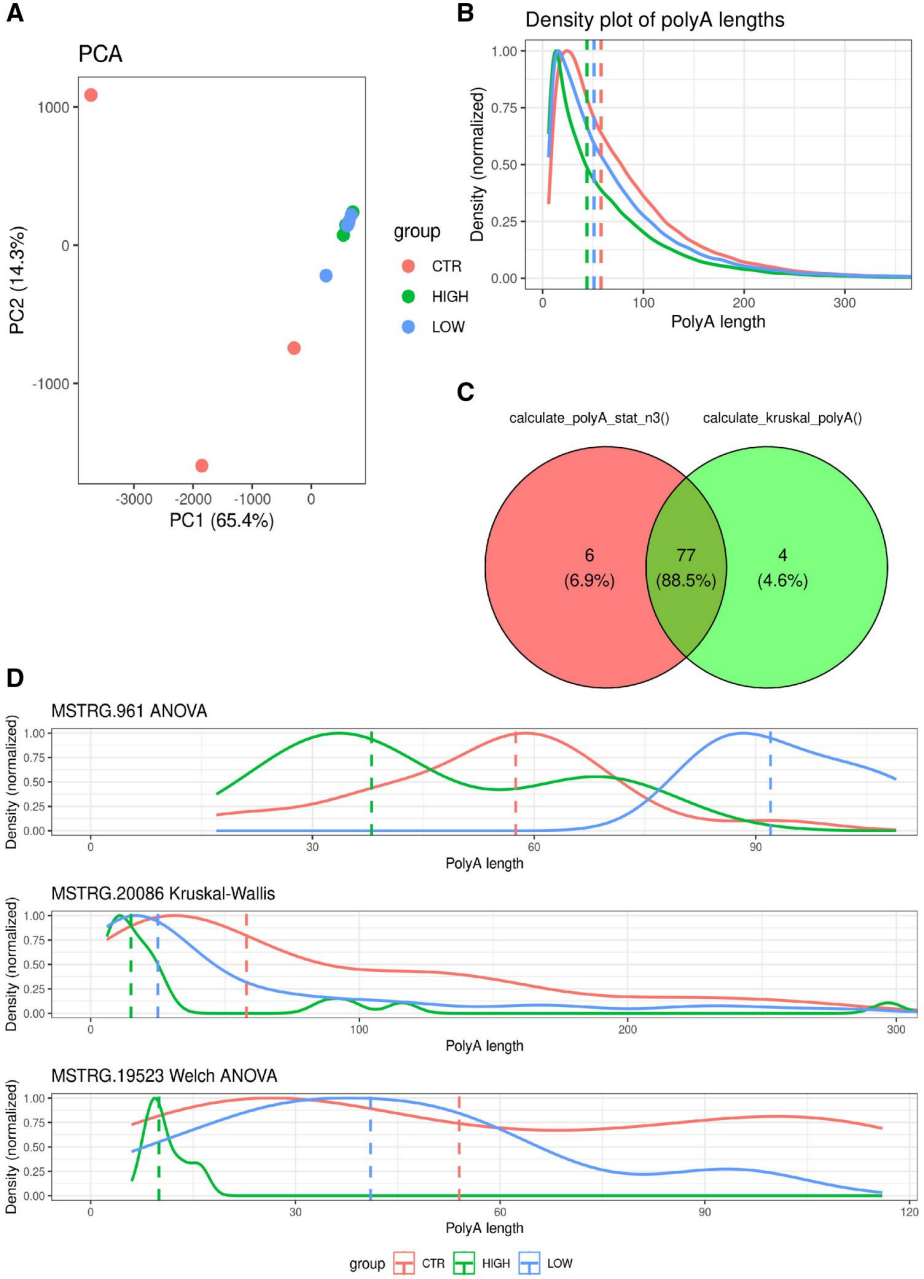
Comparison of DPGs and poly(A) tail length analysis workflow. (A) PCA plot illustrates the separation of samples based on the median poly(A) tail length. PC1 is displayed on the X-axis, and PC2 is displayed on the Y-axis. (B) Density comparison of poly(A) tail lengths among the CTR (red), LOW (blue), and HIGH (green) groups. The X-axis represents poly(A) tail lengths, and the Y-axis shows the normalized density. Vertical dashed lines indicate the median for each group. (C) Venn diagram comparing DPGs in the context of nanoplastics. The red circle represents unique DPGs identified by the *TAILcaller*::calculate_polyA_stat_n3() function. The green circle symbolizes unique DPGs identified by the *TAILcaller*::calculate_kruskal_polyA() function. The intersection (center of the diagram) denotes DPGs common to both methods. (D) Figure displays the distributions of poly(A) tail lengths and the corresponding statistical tests used, based on assumptions of normality and homogeneity of variances.

For a case study demonstrating the *TAILcaller* program, a nanoplastic project was used, where changes in poly(A) tails between three nanoplastic concentrations (CTR, LOW, and HIGH groups) were investigated. PCA analysis was performed using *TAILcaller*::PCA_polyA(), which revealed significant similarity between the HIGH and LOW samples, as well as between CTR4 and CTR5. Only samples CTR1, CTR2, and CTR3 were divergent (Fig. 2A).

An overall statistical analysis of differences between the CTR, LOW, and HIGH groups was conducted using the Kruskal-Wallis test, as implemented in the *TAILcaller*::density_plot() function. This test was automatically selected by the function after assessing the normality of distributions and homogeneity of variances. The test comparing poly(A) tail length distributions among groups showed significant differences between all groups. Specifically, tails in the CTR group were significantly longer than those in the LOW and HIGH groups, and tails in the HIGH group were significantly shorter than those in the LOW group (Fig. 2B).

Subsequently, DPGs were identified using the *TAILcaller*::calculate_polyA_stat_n3() and *TAILcaller*::calculate_kruskal_polyA() functions. The function relying on automatic test selection based on distribution and homogeneity of variances revealed 83 DPGs, while the *TAILcaller*::calculate_kruskal_polyA() function, which enforces the use of a non-parametric test, detected 81 DPGs. For both functions, 77 DPGs were detected. Additionally, six unique DPGs were detected by *TAILcaller*::calculate_polyA_stat_n3(), and four unique DPGs were detected by *TAILcaller*::calculate_kruskal_polyA() (Fig. 2C; Tables 2 and 3, available as supplementary data at *Bioinformatics Advances* online). Selected DPGs (*MSTRG.961, MSTRG.20086*, and *MSTRG.19523)* were verified, and their distributions were plotted using the *TAILcaller*::density_plot() function (Fig. 2D). Gene *MSTRG.961*, similarly to Gene *MSTRG.19523*, was not DPGs after the Kruskal-Wallis test. Nevertheless, some DPGs were detected by the *TAILcaller*::calculate_kruskal_polyA() function, but were not present among the DPGs identified by the *TAILcaller*::calculate_polyA_stat_n3() (Fig. 2C).

### 3.2 Detecting differences in poly(A) tail lengths between CTR and HIGH groups: application of *TAILcaller’s* adaptive statistical functions

**Figure 3.**
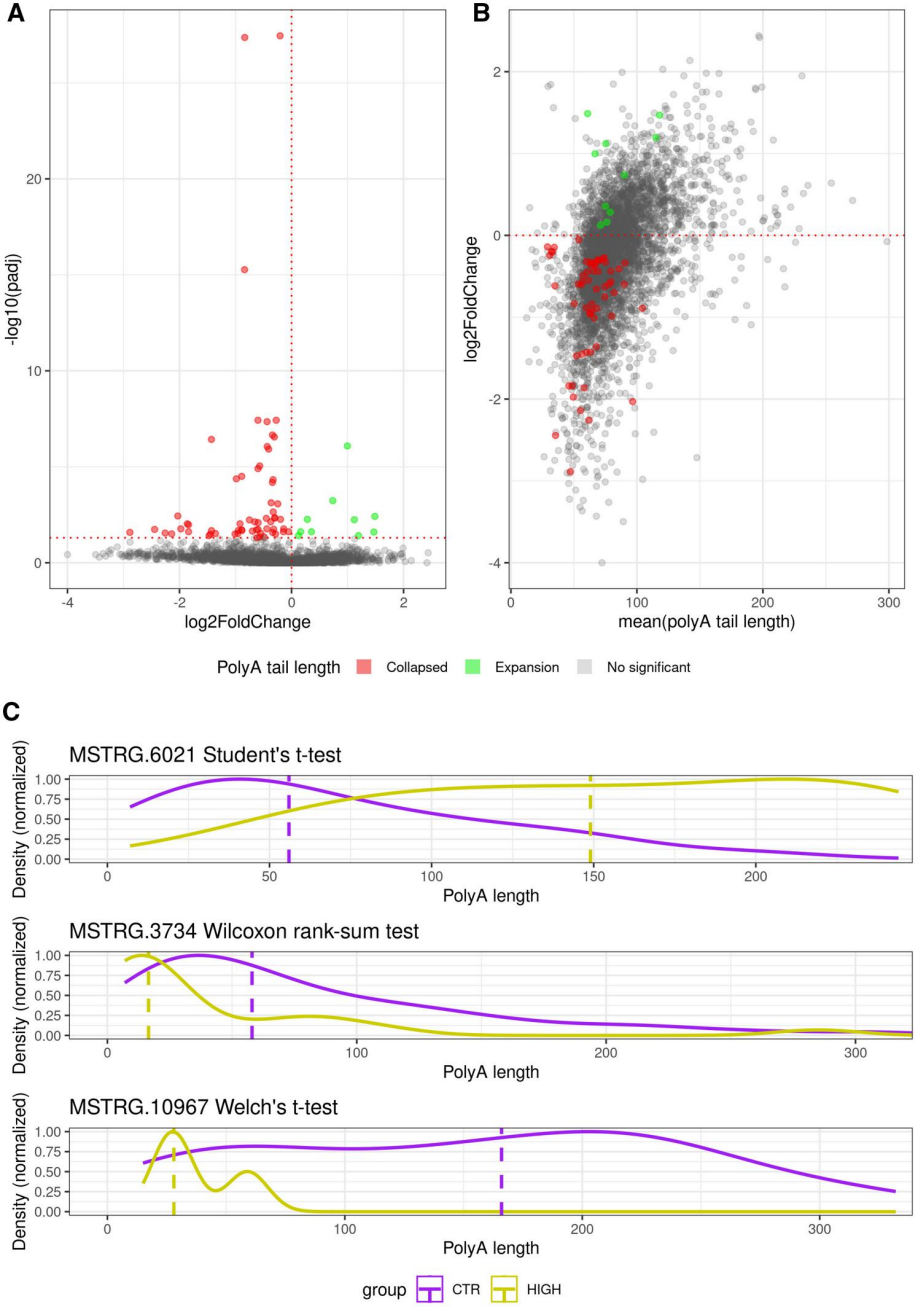
Visualization of changes in poly(A) tail lengths. (A) The volcano plot shows the relationship between log2FoldChange (X-axis) and padj (Y-axis). (B) The MA plot shows the relationship between log2FoldChange (Y-axis) and the mean poly(A) tail length (X-axis). In plots B and C, the points (polyadenylated genes) are colored: red indicates DPGs with shortened poly(A) tail lengths, green for DPGs with elongated tail lengths, and gray dots indicate genes where changes in poly(A) tail length are not statistically significant. (C) Density plots represent the distributions of poly(A) tail lengths, with the X-axis representing poly(A) tail length and the Y-axis representing normalized density. The title of each plot includes the gene ID and the statistical test used to determine the statistical significance of poly(A) tails fluctuations.

To evaluate the performance of the function for two groups, it was decided to analyze the CTR and HIGH groups, and to assess the impact of nanoplastics solely between the high concentration nanoplastic conditions. The *TAILcaller*::calculate_statistics() function identified 66 DPGs. In contrast, the *TAILcaller*::calculate_statistics_n2() function, which automatically selects the appropriate statistical test based on data distribution and homogeneity of variances, revealed 71 DPGs (Fig. 1 and Tables 4 and 5, available as supplementary data at *Bioinformatics Advances* online). It was determined that the function adapting the test to data distribution and variance homogeneity was more justified. This function revealed 61 DPGs with collapsed poly(A) tail lengths and 10 DPGs with expanded poly(A) tail lengths. Based on the results from this function, a volcano plot (*TAILcaller*::volcano_polyA()) and an MA plot (*TAILcaller*::maplot_polyA()) were generated (Fig. 3A and B).

Three DPGs were selected for detailed distribution visualization. For *MSTRG.6021*, Student’s *t*-test was employed (Shapiro−Wilk *P > .*05 for both groups; Levene *P > .*05), indicating normal distribution and homogeneous variances. For *MSTRG.3734*, the Wilcoxon rank-sum test was utilized (Shapiro−Wilk *P* ≤ .05 for at least one group), as the data did not meet the assumptions of normality. For *MSTRG.10967*, Welch’s *t*-test was used (Shapiro−Wilk *P* > .05 for both groups; Levene *P*  ≤ .05), due to normal distribution but heterogeneous variances (Fig. 3C).

### 3.3 Assessing *TAILcaller* using *R. fluitans* direct RNA sequencing data

**Figure 4.**
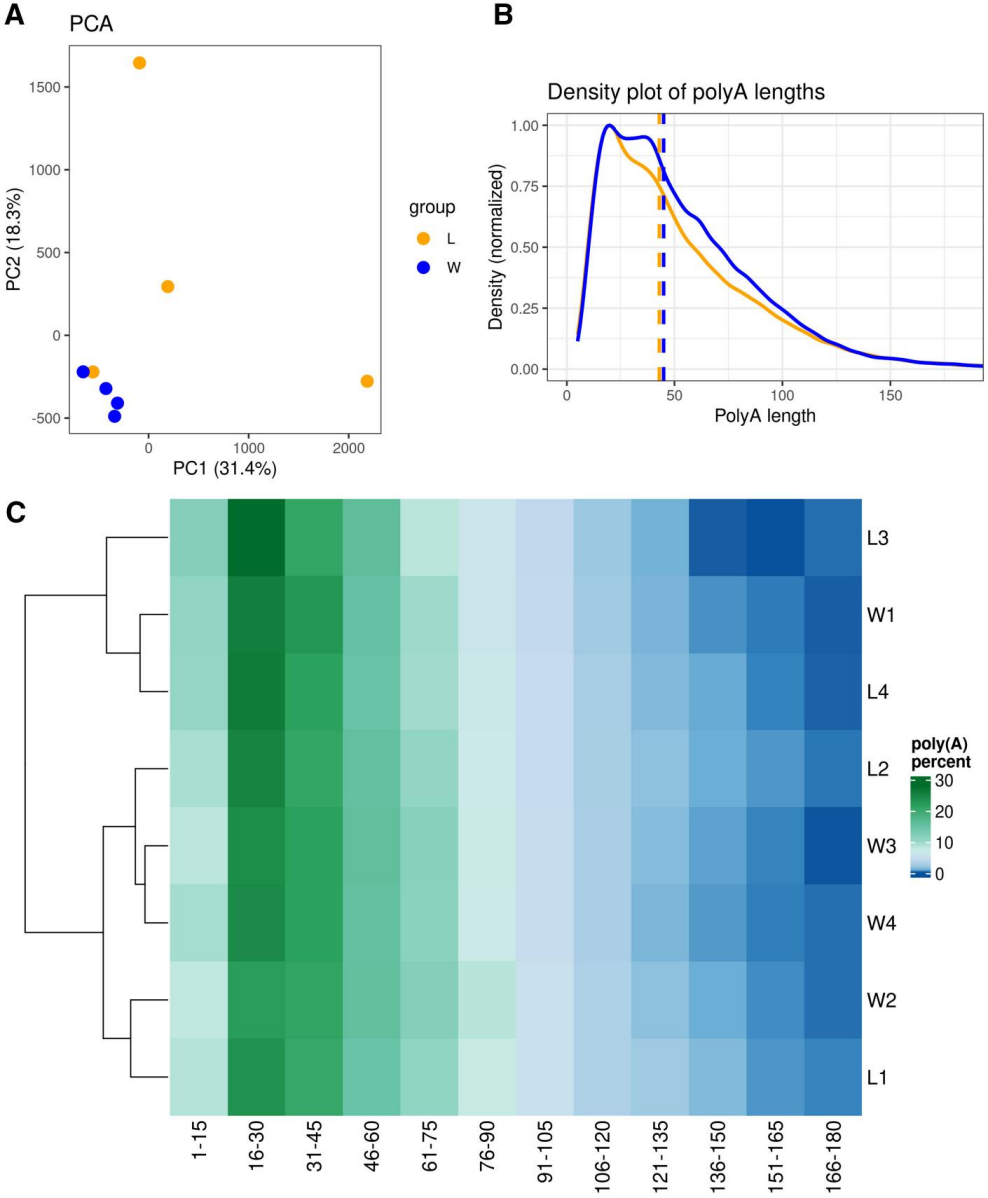
Analysis of poly(A) tail lengths in terrestrial and aquatic *R. fluitans* samples. (A) PCA illustrates the clustering of terrestrial and aquatic *R. fluitans* samples. L samples are marked in orange, and W samples are marked in blue. PC1 is represented on the X-axis, and PC2 on the Y-axis. (B) The density plot shows the distribution of poly(A) tail lengths in aquatic and terrestrial *R. fluitans*. (C) Heatmap illustrates the percentage-normalized distribution of poly(A) tails in 15-nucleotide windows.

To effectively demonstrate *TAILcaller’s* application with direct RNA sequencing data, data from our previous study were reanalyzed (Maździarz *et al.* 2024). The data were reprocessed using *dorado* for estimation and then analyzed with *TAILcaller*. PCA revealed distinct sample clustering, with sample L4 grouping with aquatic samples, while the remaining terrestrial samples (L1, L2, L3) were separated from the aquatic samples (Fig. 4A). Consistent results were obtained, showing an elongation of poly(A) tails in aquatic environments compared to terrestrial environments (*P* < .001) (Fig. 4B). Similarities between samples were revealed by hierarchical clustering, which was implemented in the *TAILcaller*::polyA_heatmap() function, used to generate a heatmap and a dendrogram. An interesting visualization alternative for lengths, in comparison to the density plot from *TAILcaller*::plot_density(), could be provided by the heatmap (Fig. 4C).

## 4 Discussion

The *TAILcaller* program is presented as a new R package designed for processing BAM files after RNA sequencing results have been basecalled by dorado. It enables users to conduct statistical analyses for both two-group comparisons and scenarios involving more than two groups.

The pipeline provided by Oxford Nanopore Technologies (https://github.com/nanoporetech/pipeline-polya-diff) is limited in several ways. First, it does not support BAM files obtained from *dorado* basecalling. Instead, it is based on the nanopolish methodology, which requires the older, unsupported guppy basecaller. Second, this program is restricted to using the Wilcoxon rank-sum test for analyzing poly(A) tail length differences, both globally and per transcript.

In contrast, *TAILcaller* has implemented a function based solely on the Wilcoxon rank-sum test, but it also features a flexible function that selects the appropriate statistical test depending on the distribution of poly(A) tail lengths and the homogeneity of variances. Furthermore, it allows for global, per-transcript, and per-gene analysis, and includes a function for visualizing results. Additionally, comparative analysis of more than two groups is made possible through a function that performs the non-parametric Kruskal-Wallis test, and again, a flexible function that chooses the test based on data distribution and homogeneity of variances.

## 5 Conclusions

*TAILcaller* is an R package that can be used to analyze differences in poly(A) tail length. It facilitates the extraction of information about poly(A) tails after basecalling with *dorado* and is used to identify the most significant changes. This package is intended to aid in the analysis of poly(A) length differences, and the results generated by this program are expected to significantly facilitate research for those studying polyadenylation and utilizing ONT.

## Supplementary Material

vbaf235_Supplementary_Data

## Data Availability

The data underlying this article are available at https://doi.org/10.6084/m9.figshare.28632515.v3.
