# Supplementary figures and images for: TAILcaller: an R package for analyzing differences in poly(A) tail length for Oxford Nanopore RNA sequencing
Source: Bioinform Adv. 2025 Sep 26;5(1):vbaf235. doi: 10.1093/bioadv/vbaf235 (PMC12527348; doi:10.1093/bioadv/vbaf235)

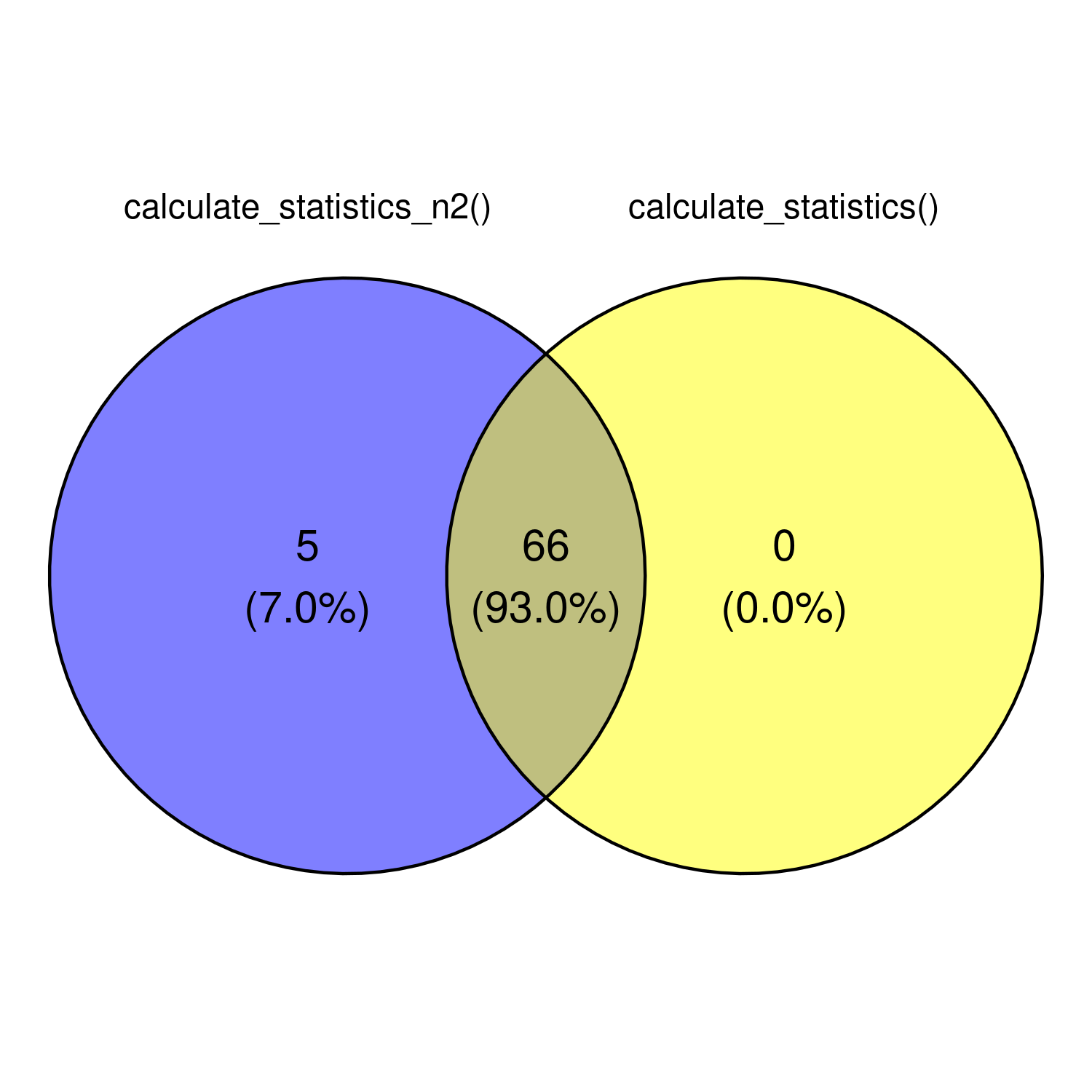

Supplement: vbaf235_Supplementary_Data [file vbaf235_supplementary_data.zip › Supplemental figure 1.png]
